# Comparison between Statistical Models and Machine Learning Methods on Classification for Highly Imbalanced Multiclass Kidney Data

**DOI:** 10.3390/diagnostics10060415

**Published:** 2020-06-18

**Authors:** Bomi Jeong, Hyunjeong Cho, Jieun Kim, Soon Kil Kwon, SeungWoo Hong, ChangSik Lee, TaeYeon Kim, Man Sik Park, Seoksu Hong, Tae-Young Heo

**Affiliations:** 1 Department of Information & Statistics, Chungbuk National University, Chungbuk 28644, Korea; qhal0324@cbnu.ac.kr (B.J.); berry8901@gmail.com (J.K.); 2 Department of Internal Medicine, Chungbuk National University College of Medicine, Chungbuk 28644, Korea; mdhjcho@gmail.com (H.C.); soonkil.kwon@gmail.com (S.K.K.); 3 Department of Internal Medicine, Chungbuk National University Hospital, Chungbuk 28644, Korea; 4 Intelligent Network Research Section, Electronics and Telecommunications Research Institute, 218 Gajeong-ro, Yuseong-gu, Daejeon 34129, Korea; swhong@etri.re.kr (S.H.); cslee2624@etri.re.kr (C.L.); tykim@etri.re.kr (T.K.); 5 Department of Statistics, Sungshin Women’s University, Seoul 02844, Korea; mansikpark@sungshin.ac.kr

**Keywords:** imbalanced data, autoencoder, machine learning, chronic kidney disease, national health screening

## Abstract

This study aims to compare the classification performance of statistical models on highly imbalanced kidney data. The health examination cohort database provided by the National Health Insurance Service in Korea is utilized to build models with various machine learning methods. The glomerular filtration rate (GFR) is used to diagnose chronic kidney disease (CKD). It is calculated using the Modification of Diet in Renal Disease method and classified into five stages (1, 2, 3A and 3B, 4, and 5). Different CKD stages based on the estimated GFR are considered as six classes of the response variable. This study utilizes two representative generalized linear models for classification, namely, multinomial logistic regression (multinomial LR) and ordinal logistic regression (ordinal LR), as well as two machine learning models, namely, random forest (RF) and autoencoder (AE). The classification performance of the four models is compared in terms of accuracy, sensitivity, specificity, precision, and F1-Measure. To find the best model that classifies CKD stages correctly, the data are divided into a 10-fold dataset with the same rate for each CKD stage. Results indicate that RF and AE show better performance in accuracy than the multinomial and ordinal LR models when classifying the response variable. However, when a highly imbalanced dataset is modeled, the accuracy of the model performance can distort the actual performance. This occurs because accuracy is high even if a statistical model classifies a minority class into a majority class. To solve this problem in performance interpretation, we not only consider accuracy from the confusion matrix but also sensitivity, specificity, precision, and F-1 measure for each class. To present classification performance with a single value for each model, we calculate the macro-average and micro-weighted values for each model. We conclude that AE is the best model classifying CKD stages correctly for all performance indices.

## 1. Introduction

Chronic kidney disease (CKD) is defined as kidney damage or the presence of a decreased glomerular filtration rate (GFR) for more than three months [1]. The National Kidney Foundation (NKF) created a guideline to help medical doctors identify the level of kidney disease and thus improve the quality of care for patients with kidney disease. The NKF presents the standard classification of kidney disease into five stages based on how well the kidneys can filter waste and excess fluid out of the blood. In the early stages, kidneys are able to filter out waste from blood. In the later stages, kidneys try to remove waste and may stop working altogether. In particular, stage 3 is separated into two stages, stage 3a and stage 3b [2].

With rising chronic non-communicable diseases, such as obesity, diabetes, and hypertension, CKD has also been increasing rapidly worldwide, and its global prevalence ranges from 8% to 18% [3]. The most common causes of CKD are diabetes and hypertension. Other problems that can damage the kidneys include glomerulonephritis, polycystic kidney disease, nephrotoxic drugs, kidney stones, and infection. CKD is associated with a significant increase in cardiovascular disease, stroke, and death [4,5]. Accordingly, early detection and effective intervention of CKD are important to slow disease progression and reduce mortality.

Kidney function screening, such as serum creatinine concentration and dipstick urine testing, is an easy and inexpensive way to detect CKD, and in Korea, it is recommended that the entire population over 40 years old be screened [1]. The National Health Screening program helps in the early detection of medical problems and contributes to reducing further medical and social costs of diseases [6]. In recent years, machine learning and deep learning methods have been used to solve the class imbalance issue [7]. Machine learning methods have been utilized to predict and classify data in the healthcare field. Various machine methods have been used to classify and predict diabetes, and magnetic resonance imaging data of the brain have been used to detect Alzheimer’s disease [8,9]. The prediction of heart disease by employing artificial neural network, decision tree, and naïve Bayes algorithms has also been proposed [10]. Moreover, a study was performed to classify and predict liver diseases by using the multilayer perceptron algorithm [11].

A similar study was conducted with 361 patients to classify different CKD stages based on the estimated GFR (eGFR) values via machine learning methods, where creatinine, gender, and age were considered as explanatory variables [12]. However, the National Health Screening dataset consists of big data that has a class imbalance problem because abnormal findings are only a small portion of the collected data [13]. A major challenge in effective healthcare data analytics is the highly imbalanced data with multiple skewed response variables due to highly unequal numbers of samples, as is the case in the present study. Highly imbalanced data may have high overall accuracy in classification and prediction results in categories with high response rates, but the sensitivity and specificity in categories with minority classes may be very low. Therefore, this study uses the entire data to classify CKD according to GFR. Building a model that considers overall accuracy and the sensitivity and specificity of each category is essential to more accurately classify highly imbalanced data and predict results.

The most commonly used method for classifying imbalanced data is to reconstruct the data through sampling techniques and apply this reconstruction to the analysis [14,15]. The oversampling technique can be used to duplicate a minority class to balance the dataset when one class of data is the minority class. Conversely, when a class of data is the majority class, undersampling can be used to balance it. However, oversampling methods may suffer from overfitting and become sensitive to overlapping between minority and majority classes, while undersampling methods easily deteriorate information about samples and classes [16]. In other words, sampling methods cannot accurately reflect the characteristics of very imbalanced data when the original data are damaged and new data are applied.

A related research showed that for imbalanced data, projecting samples from the input space on to a feature space with better classification representation between the majority and minority classes yields good classification for both classes [17]. Other studies argued that autoencoder (AE) extracts meaningful feature representation, and therefore, it is useful to develop a model for classifying imbalanced data [18,19,20,21]. On the basis of the above research, this study aims to build a model to explain the imbalanced characteristics and classify and predict more accurately by applying AE to CKD data.

In this study, we investigated the national health check data of a 134,895 samples cohort and evaluated the performance of CKD classification using generalized linear models for classification, multinomial LR and ordinal LR, and machine learning algorithms, RF and AE. It is known that there would be a bias problem in maximum likelihood estimator when modeling logistic regression with imbalanced data [22]. This is why we expected that generalized linear models would give poor classification performance. RF based on the bagging algorithm utilizes the ensemble learning technique. It would reduce the overfitting problem in decision trees and also helps reduce the variance and therefore leads to improvement in the classification performance.

The response variable of the data used in this study is the different CKD stages based on eGFR, which is a categorical variable with five stages (i.e., 1, 2, 3a, 3b, 4, and 5). In addition, this variable consists of highly imbalanced data with multiple response rate categories skewed to one side. Therefore, by constructing and comparing many machine learning models, this study identifies the factors influencing GFR through statistical models that reflect the characteristics of the ordinal response variable, with the goal of deriving the best model that more accurately classifies highly imbalanced data.

This paper is organized as follows. Section 2 describes the models and the data used in this study, including response variable, explanatory variables, and their characteristics. Section 3 presents and discusses the analysis results of comparing the classification performance between generalized linear models and machine learning algorithms. Finally, Section 4 concludes the paper with an analysis and future research directions.

## 2. Methods and Materials

### 2.1. Methods

#### 2.1.1. Multinomial Logistic Regression Model

The multinomial LR model is a generalized logistic regression model used when the response variable has three or more categories. When the logit link function, which takes the logarithmic value of the odds as the link function, is used in the generalized linear model, the resulting model is known as the multinomial LR model. The logit link function and the multinomial LR model are expressed respectively as Equations (1) and (2).

(1) $$\pi_{j}=\frac{\exp\left(\alpha +\beta_{1}x_{1}+\cdots +\beta_{p}x_{p}\right)}{1+\exp\left(\alpha +\beta_{1}x_{1}+\cdots +\beta_{p}x_{p}\right)},\;j=1,\;\cdots ,\;J-1$$

(2) $$\log\left(\frac{\pi_{j}}{1-\pi_{j}}\right)=\alpha +\beta_{1}x_{1}+\cdots +\beta_{p}x_{p}$$

where *x*_1_, …, *x_p_* are explanatory variables; *p* is the number of explanatory variables; *β*_1_, …, *β_p_* are the regression coefficients; and $\pi_{j}$ is the probability of the response variable to be in the *j*th category. Furthermore, the sum of the probabilities of categories belonging to the response variable has to be 1.

#### 2.1.2. Ordinal Logistic Regression Model

The ordinal LR model is a generalized logistic regression model used when the response variable has two categories. It is used to build models with three or more ordinal multicategory response variables. Cumulative probabilities and logits between cumulative categories can be defined and modeled as Equations (3) and (4) [23].

(3) $$P\left(Y\leq j\right)=\pi_{1}\left(x\right)+\pi_{2}\left(x\right)+\cdots +\pi_{j}\left(x\right),\;j=1,\;\cdots ,\;J$$

(4) $$\begin{array}{ll} logit\left(P\left(Y\leq j\right)\right) & =\log\left(\frac{P\left(Y\leq j\right)}{1-P\left(Y\leq j\right)}\right)=\log\left(\frac{\pi_{1}+\cdots +\pi_{j}}{\pi_{j+1}+\cdots +\pi_{J}}\right)=\alpha_{j}+\beta^{T}x,\;j\\ & =1,\;\cdots ,\;J-1 \end{array}$$

A model with cumulative logit *j* is similar to a logistic regression model that combines the categories of 1, …, *j* into one category and views the categories *j* + 1 through J as another category.

#### 2.1.3. Random Forest

Random forest (RF) as a classification tool is a technique for generating multiple decision trees through sampling with replacement and using the outcomes to derive the final result [24,25]. First, the decision tree forms a tree model, classifies data, and makes a prediction by repeating the process of dividing each variable. Decision trees are largely composed of nodes and branches and set classification criteria that develop a tree. The classification criteria used include the p-value of the chi-squared statistic, Gini Index, Entropy Index, and others. The smaller the p-value of the chi-squared statistic, the larger the impurity. The larger the Gini Index or the Entropy Index, the larger the impurity. Therefore, these indices are performed in the direction of decreasing the impurity.

**Figure 1:**
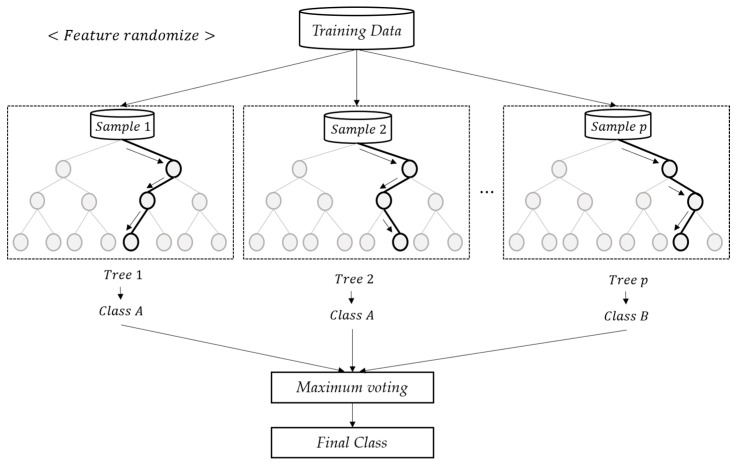
Random Forest Diagram.

As depicted in Figure 1, RF is characterized by an improved predictive performance from generating multiple decision trees through the bootstrapping technique and combining them. RF has various advantages, such as presenting unexcelled accuracy, running efficiently on large sample sizes, giving variable importance in classification, and generating internal unbiased estimates.

#### 2.1.4. Autoencoder

**Figure 2:**
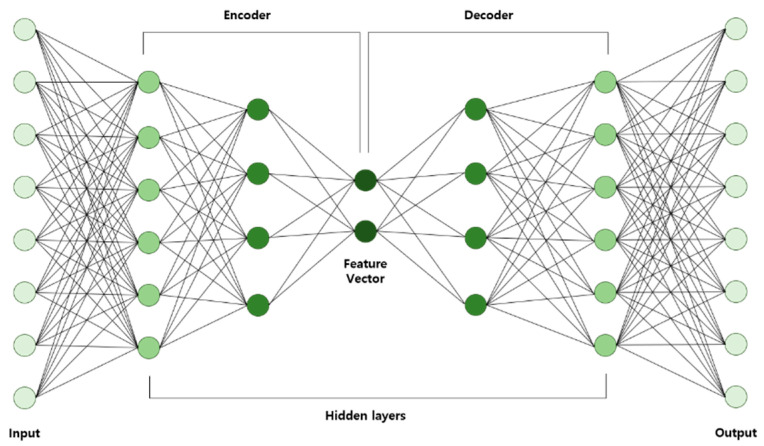
Autoencoder Diagram.

AE is a multilayer neural network structure in which the number of nodes in the input layer is equal to the nodes in the output layer. The goal of AE learning is to make the output node value equal to the input node value. A basic AE structure is shown in Figure 2. It has the structural feature in which the number of nodes from the input layer decreases on their way to the output layer and then increases at a specific point before reaching the output layer. Many different terms are used to describe the specific point, such as code, latent variable, feature vector, and hidden representation. The part where the number of nodes is reduced from the input layer is called an encoder, and the part where the number of nodes is increased to the output layer is called a decoder. This structural feature, in particular, makes it possible to perform hierarchical feature extractions or dimension reductions in the encoder part. When an AE is used to extract features, only the portion between the input layer and the middle layer is cut out. Deep learning neural networks can be configured by adding a fully connected neural network or the softmax layer behind the cutout. When training with the constructed deep learning neural network using the encoder part of the AE, both the input and output parts are used [26].

A stacked AE (SAE) is a special type of AE consisting of several layers of sparse AE where the output of each hidden layer is linked to the input of the successive hidden layer. SAE trains the hidden layer through an unsupervised learning algorithm and then fine-tunes the training with a supervised method. The three key steps of SAE are as follows [26].

- AE is trained using input data and then the learned data are acquired.
- Learned data from the previous layer are used continuously as input for the next layer until the training is completed.
- Once all the hidden layers are trained, the backpropagation algorithm is used to minimize the cost function and the weights are updated with the training set to achieve fine-tuning.

The advantage of SAE is that it extracts much detailed information from the raw data, thus providing better features from all explanatory variables. This characteristic of SAE eventually improves the accuracy in model performance [26].

#### 2.1.5. Classification Performance

In this section, two generalized linear models for classification, namely, multinomial LR and ordinal LR models, as well as two machine learning method models, namely, RF technique and AE, were applied and compared to identify the model that best classifies CKD stages of highly imbalanced data.

In traditional model assessment, the models are fitted once by using the original training data. Thanks to recent advances in computing power, cross validation (CV) is necessarily conducted to select the better model because it can provide researchers information about the variability of fitted models. There are several CV methods, such as the hold-out method (the validation set approach), leave-one-out CV (LOOCV), and K-fold CV. LOOCV is a special of K-fold CV in which K is set to equal the sample size n.

**Figure 3:**
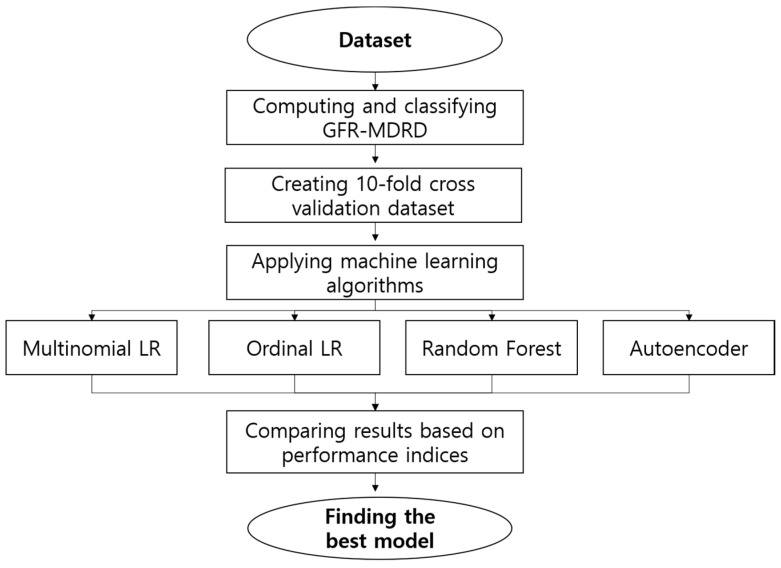
Methodology and performance workflow.

LOOCV is clearly computationally expensive, especially for AE. Hence, as a means to test the data obtained from this study, the K-fold CV was utilized. K-fold CV tested and evaluated the data collected, which were split into k-subsets to guarantee the reliability for classification performance. A 10-fold CV was applied in this study. Figure 3 presents the methodology and performance workflow of this study.

To compare the performance of each model, a confusion matrix was calculated for each test set, and the accuracy, sensitivity, specificity, precision, and F1-Measure of the models were calculated and compared. Accuracy, sensitivity, specificity, precision, and F1-Measure were calculated using the mean value of 10 confusion matrices. Specificity corresponds to the ratio of accurate prediction in the case where the classification of the response variable is higher than the reference level. Sensitivity corresponds to the ratio of accurate prediction in the case where the classification of CKD stages is lower than the reference level.

Sensitivity and specificity are considerably more important than accuracy when evaluating the performance on highly imbalanced data because the imbalanced dataset is either dominated with positive or negative cases.

Accuracy = (TP + TN)/(TP + FP + TN + FN) Sensitivity = TP/(TP + FN) Specificity = TN/(TN + FP) Precision = TP/(TP + FP) F1-Measure = 2× (Sensitivity × Precision)/(Sensitivity + Precision)

where TP is the number of true positive classification cases, TN is the number of true negative classification cases, FP is the number of false positive classification cases, and FN is the number of false negative classification cases.

For multiclass classification performance of 10-fold CV, 10 models are built on the basis of 10 CV datasets, yielding 10 confusion matrices. To calculate a single value of accuracy, sensitivity, specificity, precision, and F1-Measure, the averages of the 10 values of performance measure were obtained.

### 2.2. Materials

#### 2.2.1. Dataset

**Figure 4:**
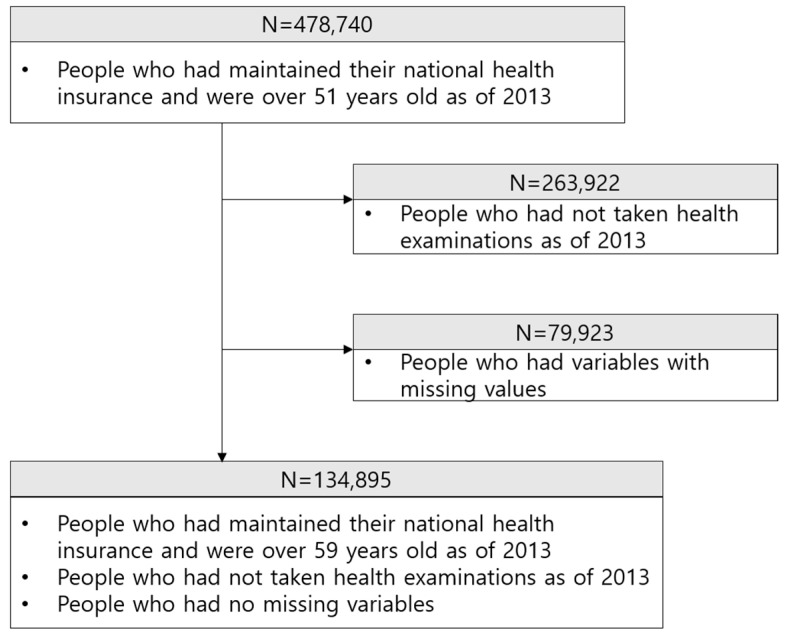
Data workflow.

National Health Insurance Corporation (NHIC) in Korea provides Koreans with the National Health Insurance Service. NHIC collects several types of personal medical data and manages a database according to Article 1 of the “Act on Provision and Utilization of Public Data.” This study utilized the health examination cohort database managed by the NHIC. Data on 478,740 persons, selected through a simple random sampling of 10% of all persons aged 51 years and older who had maintained health insurance status as of 2013 were used. Figure 4 shows a schematic of the study subjects. Among the total 478,740 possible subjects, 214,818 samples who had received general health examinations administered by the NHIC were chosen initially. The 263,922 people who had not taken health examinations as of 2013 were excluded from the dataset because they did not have medical information at that time. After the samples with missing information or variables were eliminated, the final group of study subjects included 134,895 people. The data that support the findings of this study are available from the National Health Insurance Sharing Service (https://nhiss.nhis.or.kr).

#### 2.2.2. Description of Variable

The explanatory and response variables of this study are shown in Table 1 below. Categorical explanatory variables include income deciles, type of disability, gender, and smoking status, while continuous explanatory variables include age, fasting blood sugar, body mass index (BMI), systolic pressure, diastolic pressure, serum creatinine, gamma glutamyl transpeptidase (GTP), high-density lipoprotein (HDL) cholesterol, low-density lipoprotein (LDL) cholesterol, hemoglobin, aspartate amino-transferase (AST), alanine amino-transferase (ALT), total cholesterol, triglycerides, and waist measure. In addition, GFR combined with the Modification of Diet in Renal Disease (GFR-MDRD) was used as one of the response variables, which are described in detail in Section 3.1.

#### 2.2.3. Stages of CKD

The GFR used as the response variable in this study is categorized into six stages, and the calculation formula for GFR using the MDRD method is as follows:

(5) $$\begin{array}{ll} GFR_{MDRD}= & 186\times {\left(Creatinine/88.4\right)}^{-1.154}\times {\left(Age\right)}^{-0.203}\\ & \;\times \{\begin{array}{c} 1,\;if\;male\;\\ 0.742,\;if\;female \end{array}\times \{\begin{array}{c} 1.210,\;if\;black\;\\ 1,\;otherwise\; \end{array} \end{array}$$

The result of Equation (5) was technically divided into five stages according to the criteria in Table 2. This study regards stages 3A and stage 3B as individual stages and used six CKD stages based on eGFR as the response variable.

#### 2.2.4. The Property and Treatment of Highly Imbalanced Data

A classification problem on highly imbalanced data predominantly occurs in the real world. Highly imbalanced data have an extremely skewed distribution, in that the number of observations belonging to one class is significantly lower than those belonging to other classes. The problem hinders analytic models from sufficiently training and learning the feature of the minority class. In this situation, the predictive model developed using conventional machine learning algorithms could be biased and inaccurate.

Sampling technique is considered as one of the approaches for solving such class imbalanced problems. The main idea of this technique is to create a balanced class distribution. Various sampling techniques have their pros and cons. For example, random oversampling increases the number of cases in the minority class by replicating these cases at random, and undersampling is used to balance a dataset with the minority class when a class of data is the overrepresented majority class. Unlike the undersampling technique, random oversampling does not lead to any information loss. However, it may result in overfitting because it replicates minority class events.

In this study, we tried to avoid using sampling techniques to find the best classification model on highly imbalanced data and instead compared generalized linear models to recent machine learning algorithms for original data.

#### 2.2.5. Preprocessing

Data preprocessing was initially conducted via one-hot encoding for categorical variables and scaling for continuous variables. Resampling methods were not utilized so that the performance of AE for the original imbalance dataset could be confirmed.

We used R 3.6.2 as a statistical analysis tool and packages such as “nnet,” “ordinal,” “randomForest,” and “h2o” to build models, multinomial LR, ordinal LR, RF, and AE, respectively. For RF, the number of variables randomly sampled as candidates at each split was set to $\sqrt{19}$, and the number of trees to grow was set to 10.

In the first step of AE, we treated our input data as labeled with the same input values. Next, the network was forced to learn the identity through a nonlinear and reduced representation for half of each training data in order for a neural network to obtain convergence. This process is called unsupervised, layer-wise pretraining of supervised tasks. The second step of AE was to fine-tune the learned model for the other half of training data to build a final AE model. The hidden layer was set to (50, 20, 6, 20, 50) for both unsupervised and supervised tasks.

## 3. Results and Discussion

The results presented in this section are as follows. Section 3.1 presents the baseline characteristics according to the six stages of CKD based on eGFR. Section 3.2 provides the results of the four built models to classify the response variable into multiple classes. It likewise compares the classification performance of the four models in terms of accuracy, sensitivity, specificity, precision, and F1-Measure so we can choose the best classification model. Finally, Section 3.3 compares the results of this study with those of a similar study and discusses the applicability of AE to other medical fields.

### 3.1. Baseline Characteristic of Variables

This section discusses the baseline characteristics of all explanatory variables by each class of response variable. Table 3 shows the mean, standard deviation, and frequency of each variable according to the six categories of GFR-MDRD.

A look at the frequencies of the six categories of GFR-MDRD in Table 3 reveals a sharp decrease in frequency in the high-risk group compared with that in the normal group, and in the most at-risk groups of <15 and 15–29, the frequencies are very small at 0.33% and 0.15%, respectively. These figures indicate the need for caution when interpreting classification performance, such as accuracy, sensitivity, and so on. Creatinine levels tend to increase rapidly as we move from the normal to the risk groups, and age and the proportion of women are higher.

### 3.2. Results

Table 4 shows the classification results obtained by applying the generalized linear models and machine learning models presented above to the different CDK stages. The average accuracies of the four models are all high at 0.8244, 0.9682, 0.9948, and 0.9958 for multinomial LR, ordinal LR, RF, and AE, respectively. The average accuracy for the two machine learning models is slightly higher than that for the generalized linear models. However, when it comes to the performance interpretation on a highly imbalanced dataset, average sensitivity and average specificity should be considered as key criteria. A close look at the average sensitivity of stages 4 and 5, minority classes, and interesting categories reveals that multinomial LR and RF present poor sensitivity given that the sensitivity of stage 4 shows 0.0195 and 0.2771, respectively. Meanwhile, the sensitivity of stage 5 for multinomial LR and RF is also not enough to signify if such algorithms are good models.

On the other hand, ordinal LR and AE show overwhelming performance figures, especially in their average sensitivity for stages 4 and 5. The average sensitivity for ordinal LR (0.9797, 0.9746, 8363, 0.8265, 0.8319, 0.9818) is higher than that for multinomial LR (0.9217, 0.8482, 0.0113, 0.0091, 0.0195, 0.6249). Therefore, the average sensitivity for AE (0.9976, 0.9965, 0.9905, 0.9223, 0.8462, 0.9818) is definitely better than that for RF (0.9998, 0.9996, 0.9792, 0.7578, 0.2771, 0.6585), except for stages 1 and 2.

When comparing the average values of accuracy, sensitivity, and specificity of the four models applied in this study, the AE has the highest accuracy of 0.9958, with the average sensitivity of 0.9976, 0.9965, 0.9905, 0.9223, 0.8462, and 0.9818, indicating that it has the highest value in the risk group (30–44, 15–29, <15) among the four models. Therefore, AE performance is the best among the four models in classifying the CKD stages of highly imbalanced data.

Table 4 also gives the standard deviation for the 10-fold CV confusion matrix. A comparison of the standard deviation for classification performance, such as accuracy, sensitivity, specificity, precision, and F1-Measure between algorithms, confirms that AE shows a lower value of standard deviation than the standard deviation of multinomial LR, ordinal LR, and RF.

The incomplete F1-Measure from multinomial LR and RF seems to be strange. This phenomenon is derived from that fact that F1-Measure is calculated by averaging the F1-Measure from 10 confusion matrices. If both sensitivity and precision are 0 in a certain confusion matrix, then F1-Measure is not available because the denominator is 0. This fact also provides us evidence that multinomial LR and RF perform poorly in modeling for one or more specific CV sets.

The model comparison of classification performance indices, such as accuracy, sensitivity, specificity, precision, and F1-Measure, shows that machine learning algorithms, RF and AE, have better performance than do multinomial LR and ordinal LR. With respect to sensitivity, ordinal LR and AE present better performance than do multinomial LR and RF. This result is based on the sensitivity for the minority class in stages 4 and 5. All these results from the statistical modeling suggest that AE is the best model for dealing with highly imbalanced data without resampling techniques.

AE algorithm based on artificial neural network gives the best classification result when compared with the other three techniques in classifying CKD into six stages, as shown below. The accuracy of AE is 99.58%.

- Stage 1 patients: sensitivity 99.76%, specificity 99.79%, precision 99.64%, and F1-Measure 99.70%
- Stage 2 patients: sensitivity 99.65%, specificity 99.73%, precision 99.80%, and F1-Measure 99.72%
- Stage 3A patients: sensitivity 99.05%, specificity 99.89%, precision 99.97%, and F1-Measure 98.51%
- Stage 3B patients: sensitivity 92.23%, specificity 99.96%, precision 93.77%, and F1-Measure 92.96%
- Stage 4 patients: sensitivity 84.62%, specificity 99.98%, precision 85.94%, and F1-Measure 84.66%
- Stage 5 patients: sensitivity 98.18%, specificity 99.99%, precision 98.21%, and F1-Measure 98.18%

**Figure 5:**
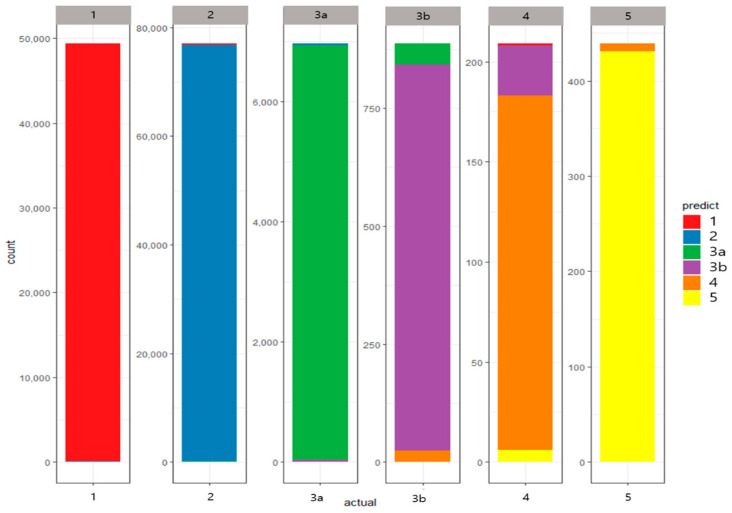
Confusion matrix for autoencoder (AE) based on 10-fold cross validation.

In addition, a confusion matrix for AE is calculated by the sum of confusion matrices from 10-fold CV in Figure 5. The areas of orange and yellow color for stages 4 and 5 indicate, respectively, the correctly classified cases for each stage. Figure 5 graphically confirms the high sensitivity for each stage presented above, indicating that many cases are correctly classified, and the number of diagonal elements occupies most cases in the confusion matrix from AE.

Specifically, the values of sensitivity for stages 3B and 4 are 92.23% and 84.62%, respectively. These figures are lower than the other values of sensitivity because of the rare cases in stages 3B (0.66%) and 4 (0.15%). Although this phenomenon often occurs in an imbalanced dataset, we can interpret that the sensitivities for stages 3B (92.23%) and 4 (84.62%) show good performance.

Table 5 lists the macro-averaged and micro-weighted sensitivity, precision, and F1-Measure for each model. This step is conducted to combine the per-class sensitivity, precision, and F1-Measure into a single value. Macro values are calculated through the arithmetic mean of per-class values. On the other hand, to calculate micro values, the sensitivity, precision, and F1-Measure of each class are weighted by the number of samples from that class.

Given that some cells of the F1-Measure are not available for multinomial LR and RF, we can confirm that the micro F1-Measures for two models are vacant. Similar to the result from Table 4, it can be confirmed from Table 5 that AE shows superior classification performance over other models on the basis of the macro and micro values.

### 3.3. Discussion

For logistic regression to show good classification performance, the number of cases of the categorical response variables has to be evenly distributed. When logistic regression is applied to unbalanced data, the unbiasedness of coefficients cannot be guaranteed. From the analysis result of this work, the classification performance for minority classes is confirmed to be relatively lower than the performance measure from AE. Clearly, the performance of ordinal LR is better than that of multinomial LR. A possible explanation for this outcome is that ordinal LR utilizes more samples to build a model than does multinomial LR. The fact that the response variables have an ordered category may be a reason for this as well. However, ordinal LR presents a less balanced performance for both majority and minority classes than does AE.

The reason this study used AE for modeling on imbalanced data is to check whether feature extraction, an outstanding advantage of AE, is effective for minority classes. The results confirm that AE shows excellent performance in both majority and minority classes. This study attempts to build a model by using raw data without the use of sampling techniques for imbalanced data. The result can be interpreted as meaning that AE is a model that can be free from the problem of data distortion of the sampling technique. One of the characteristics of medical data is that the data are highly imbalanced because interesting events tend to happen rarely. For these reasons, AE can be widely applied to a variety of medical research fields.

One reason AE shows better classification performance in this study may be due to the large number of samples. A related study classifying different CKD stages based on eGFR values shows that probabilistic neural network presents better performance than other machine learning algorithms, such as multilayer perceptron algorithm, support vector machine, and radial basis function. The dataset used consisted of 361 Indian patients with CKD and contained 25 variables (11 numerical and 14 categorical) [12]. The sample size is substantially less than that of our research, which had 134,895 samples. Therefore, a possibility exists that classification performance may be affected by the total sample size.

## 4. Conclusions

Several studies have confirmed that AE has excellent performance in feature extraction. Results of this study reveal that this advantage of AE enables it to learn the characteristics of minority samples in highly imbalanced data. With most medical data having the form of imbalanced data, the possibility of applying AE in various medical diagnoses is very strong. For example, AE can be utilized to detect rare symptoms via ECG or EEG signals in the form of sound data. The value of AE use will also be high in the field of disease diagnosis using X-ray, CT, and MRI, which are image data types.

This study did not perform a comparison between the models built with the dataset using the resampling technique and models built using the original data. This decision was made on the basis of the large number of samples. Contrarily, there are many small sample data in medical fields. Therefore, it would be very meaningful to study the effectiveness of the recently suggested sampling techniques.

## Figures and Tables

**Table 1:** Variable description used in the analysis.

Variable	Type	Class	Description	
CKD_eGFR	Categorical	Target	eGFR using MDRD	1, 2, 3A, 3B, 4, 5
INCOME_DECILES	Categorical	Predictor	Income deciles	0, 1, 2, …, 10
DISABILITY	Categorical	Predictor	Type of disability	0: Not disabled (Normal) 1: Mild 2: Severe
SEX	Categorical	Predictor	Gender	1: Male 2: Female
SMOKING_STATUS	Categorical	Predictor	Smoking status	1: No 2: Past 3: Current
AGE	Numerical	Predictor	Age	-
BLDS	Numerical	Predictor	Fasting blood sugar	mg/dL
BMI	Numerical	Predictor	Body mass index	Weight(kg)/(Height × Height(m^2^))
BP_HIGH	Numerical	Predictor	Systolic pressure	mmHg
BP_LWST	Numerical	Predictor	Diastolic pressure	mmHg
CREATININE	Numerical	Predictor	Serum Creatinine	mg/dL
GAMMA_GTP	Numerical	Predictor	Gamma Glutamyl Transpeptidase (GTP)	U/L
HDL_CHOLE	Numerical	Predictor	High Density Lipoprotein (HDL) Cholesterol	mg/dL
HMG	Numerical	Predictor	Hemoglobin	g/dL
LDL_CHOLE	Numerical	Predictor	Low Density Lipoprotein (LDL) Cholesterol	mg/dL
SGOT_AST	Numerical	Predictor	AST (Aspartate Amino-Transferase)	U/L
SGPT_ALT	Numerical	Predictor	ALT (Alanine Amino-Transferase)	U/L
TOT_CHOLE	Numerical	Predictor	Total Cholesterol	mg/dL
TRIGLYCERIDE	Numerical	Predictor	Triglycerides	mg/dL
WAIST	Numerical	Predictor	Waist measure	cm

**Table 2:** Stages of glomerular filtration rate (GFR).

**Stage**	**GFR**	**Description**
1	≥90	Normal or high
2	60~89	Mildly decreased
3A	45~59	Mildly to moderately decreased
3B	30~44	Moderately to severely decreased
4	15~29	Severely decreased
5	<15	Kidney Failure

**Table 3:** Baseline characteristic of Variables.

Variable		GFR-MDRD (Mean ± SD or N (%))						
		Total	≥90 (1)	60~89 (2)	45~69 (3a)	30~44 (3b)	15~29 (4)	<15 (5)
N (%)		134,895	49,378 (36.6)	77,018 (57.09)	6964 (5.16)	887 (0.66)	209 (0.15)	439 (0.33)
INCOME_DECILES	0	651	9447 (33.36)	17,157 (60.59)	1382 (4.88)	188 (0.66)	36 (0.13)	105 (0.37)
	1	9714	227 (34.87)	349 (53.61)	55 (8.45)	8 (1.23)	4 (0.61)	8 (1.23)
	2	8331	3321 (34.19)	5622 (57.88)	649 (6.68)	78 (0.8)	19 (0.2)	25 (0.26)
	3	9405	3151 (37.82)	4627 (55.54)	463 (5.56)	50 (0.6)	19 (0.23)	21 (0.25)
	4	8643	3715 (39.5)	5095 (54.17)	499 (5.31)	63 (0.67)	15 (0.16)	18 (0.19)
	5	9793	3374 (39.04)	4749 (54.95)	427 (4.94)	54 (0.62)	9 (0.1)	30 (0.35)
	6	11,120	3877 (39.59)	5338 (54.51)	480 (4.9)	52 (0.53)	11 (0.11)	35 (0.36)
	7	12,848	4465 (40.15)	5970 (53.69)	548 (4.93)	64 (0.58)	20 (0.18)	53 (0.48)
	8	15,444	5054 (39.34)	7082 (55.12)	597 (4.65)	74 (0.58)	11 (0.09)	30 (0.23)
	9	20,631	5720 (37.04)	8745 (56.62)	815 (5.28)	105 (0.68)	23 (0.15)	36 (0.23)
	10	28,315	7027 (34.06)	12,284 (59.54)	1049 (5.08)	151 (0.73)	42 (0.2)	78 (0.38)
DISABLED_TYPE	Normal	134,067	49,146 (36.66)	76,552 (57.1)	6874 (5.13)	861 (0.64)	204 (0.15)	430 (0.32)
	Mild	606	173 (28.55)	350 (57.76)	61 (10.07)	19 (3.14)	3 (0.5)	0 (0)
	Severe	222	59 (26.58)	116 (52.25)	29 (13.06)	7 (3.15)	2 (0.9)	9 (4.05)
SEX	Male	77,118	28,095 (36.43)	44,944 (58.28)	3229 (4.19)	488 (0.63)	125 (0.16)	237 (0.31)
	Female	57,777	21,283 (36.84)	32,074 (55.51)	3735 (6.46)	399 (0.69)	84 (0.15)	202 (0.35)
SMOKING_STATUS	No	82,346	29,766 (36.15)	46,724 (56.74)	4857 (5.9)	585 (0.71)	133 (0.16)	281 (0.34)
	Past	30,885	10,538 (34.12)	18,580 (60.16)	1393 (4.51)	214 (0.69)	54 (0.17)	106 (0.34)
	Current	21,664	9074 (41.89)	11,714 (54.07)	714 (3.3)	88 (0.41)	22 (0.1)	52 (0.24)
AGE		60.87 ± 8	58.75 ± 6.67	61.49 ± 8.2	67.5 ± 8.29	70.7 ± 9.03	69.06 ± 8.91	62.36 ± 7.94
BLDS		102.53 ± 24.44	101.81 ± 24.19	102.45 ± 23.69	106.5 ± 29.13	113.19 ± 39.61	114.91 ± 47.65	106.52 ± 30.7
BMI		24.01 ± 2.92	23.77 ± 2.93	24.11 ± 2.88	24.56 ± 3.01	24.66 ± 3.17	24.17 ± 3.53	24.09 ± 2.92
BP_HIGH		124.6 ± 14.51	124.11 ± 14.41	124.59 ± 14.4	127.16 ± 15.43	128.45 ± 16.51	131.88 ± 18.23	130.11 ± 16.64
BP_LWST		76.89 ± 9.51	76.86 ± 9.57	76.9 ± 9.43	77.01 ± 9.83	75.95 ± 10	77.03 ± 10.72	77.79 ± 9.93
CREATININE		0.92 ± 0.55	0.74 ± 0.14	0.96 ± 0.15	1.21 ± 0.18	1.65 ± 0.27	2.58 ± 0.56	8.2 ± 5.17
GAMMA_GTP		36.46 ± 49.03	37.91 ± 55.22	35.79 ± 45.42	33.84 ± 40.86	37.98 ± 53.29	31.73 ± 29.1	31.32 ± 29.53
HDL_CHOLE		53.37 ± 15.84	54.49 ± 15.75	52.96 ± 15.82	50.89 ± 16.4	47.05 ± 12.89	47.33 ± 14	53.06 ± 14.39
HMG		14.08 ± 1.44	14.08 ± 1.38	14.14 ± 1.43	13.55 ± 1.55	12.68 ± 1.69	11.49 ± 1.72	13.21 ± 1.97
LDL_CHOLE		117.67 ± 34.89	116.19 ± 34.99	118.98 ± 34.5	116.08 ± 37.08	105.83 ± 37.7	101.01 ± 33.05	111.74 ± 36.33
SGOT_AST		26.39 ± 15.74	26.34 ± 15.07	26.45 ± 16.39	26.58 ± 13.09	25.6 ± 16.03	23.33 ± 11	23.05 ± 8.9
SGPT_ALT		24.55 ± 17.84	24.74 ± 18.19	24.59 ± 17.81	23.42 ± 15.96	21.7 ± 18.24	18.85 ± 12.85	20.7 ± 10.24
TOT_CHOLE		197.16 ± 37.23	196.16 ± 36.48	198.24 ± 37.19	195.01 ± 40.87	184.52 ± 42.64	176.32 ± 39.99	190.56 ± 40.51
TRIGLYCERIDE		133.14 ± 81.97	130.33 ± 83.11	133.9 ± 81.16	141.36 ± 79.96	160.87 ± 98.44	136.99 ± 61.22	128.22 ± 76.73
WAIST		82.04 ± 8.21	81.26 ± 8.15	82.33 ± 8.17	83.81 ± 8.38	85.45 ± 8.52	84.33 ± 9.68	82.39 ± 8.37

**Table 4:** Classification performance table (average and standard deviation for 10-fold CV confusion matrix).

Method	Class	Accuracy (S.D.)	Sensitivity (S.D.)	Specificity (S.D.)	Precision (S.D.)	F1-Measure (S.D.)
**Multinomial LR**	**1**	0.8244 (0.0153)	0.9217 (0.0191)	0.8764 (0.0240)	0.8125 (0.0306)	0.8633 (0.0205)
	**2**		0.8482 (0.0176)	0.8025 (0.0157)	0.8510 (0.0122)	0.8496 (0.0142)
	**3A**		0.0113 (0.0094)	0.9968 (0.0024)	0.1426 (0.0771)	-
	**3B**		0.0091 (0.0139)	0.9969 (0.0034)	0.0000 (0.0000)	-
	**4**		0.0195 (0.0345)	0.9977 (0.0028)	0.0105 (0.0184)	-
	**5**		0.6249 (0.3307)	0.9960 (0.0029)	0.3814 (0.1947)	-
**Ordinal LR**	**1**	0.9682 (0.0010)	0.9797 (0.0019)	0.9982 (0.0011)	0.9796 (0.0018)	0.9797 (0.0013)
	**2**		0.9746 (0.0014)	0.9651 (0.0022)	0.9738 (0.0016)	0.9742 (0.0010)
	**3A**		0.8363 (0.0101)	0.9916 (0.0006)	0.8440 (0.0099)	0.8401 (0.0077)
	**3B**		0.8265 (0.0345)	0.9989 (0.0003)	0.8332 (0.0381)	0.8293 (0.0297)
	**4**		0.8319 (0.1167)	0.9997 (0.0001)	0.8314 (0.0555)	0.8269 (0.0655)
	**5**		0.9818 (0.0209)	0.9999 (0.0001)	0.9802 (0.0240)	0.9807 (0.0118)
**RF**	**1**	0.9948 (0.0015)	0.9998 (0.0002)	0.9997 (0.0002)	0.9995 (0.0004)	0.9997 (0.0003)
	**2**		0.9996 (0.0003)	0.9969 (0.0013)	0.9976 (0.0010)	0.9986 (0.0006)
	**3A**		0.9792 (0.0084)	0.9977 (0.0008)	0.9594 (0.0136)	0.9692 (0.0104)
	**3B**		0.7578 (0.0799)	0.9989 (0.0003)	0.8156 (0.0475)	0.7848 (0.0618)
	**4**		0.2771 (0.1445)	0.9998 (0.0002)	0.6170 (0.2700)	-
	**5**		0.6585 (0.1342)	0.9998 (0.0001)	0.9270 (0.0549)	0.7629 (0.1010)
**AE**	**1**	0.9958 (0.0005)	0.9976 (0.0010)	0.9979 (0.0005)	0.9964 (0.0009)	0.9970 (0.0005)
	**2**		0.9965 (0.0008)	0.9973 (0.0009)	0.9980 (0.0007)	0.9972 (0.0005)
	**3A**		0.9905 (0.0039)	0.9989 (0.0004)	0.9797 (0.0063)	0.9851 (0.0032)
	**3B**		0.9223 (0.0281)	0.9996 (0.0001)	0.9377 (0.0257)	0.9296 (0.0183)
	**4**		0.8462 (0.1181)	0.9998 (0.0002)	0.8594 (0.0762)	0.8466 (0.0658)
	**5**		0.9818 (0.0179)	0.9999 (0.0001)	0.9821 (0.0231)	0.9818 (0.0178)

**Table 5:** Classification performance table (macro and micro values).

Method	Macro Sensitivity	Micro Sensitivity	Macro Precision	Micro Precision	Macro F1-Measure	Micro F1-Measure
**Multinomial LR**	0.4058	0.8244	0.3663	0.7919	0.8565	-
**Ordinal LR**	0.9051	0.9682	0.9070	0.9681	0.9052	0.9681
**RF**	0.7787	0.9948	0.8860	0.9943	0.9030	-
**AE**	0.9558	0.9958	0.9589	0.9958	0.9562	0.9958
